# Phosphorylated (pT371)TRF1 is recruited to sites of DNA damage to facilitate homologous recombination and checkpoint activation

**DOI:** 10.1093/nar/gkt775

**Published:** 2013-08-30

**Authors:** Megan McKerlie, John R. Walker, Taylor R. H. Mitchell, Florence R. Wilson, Xu-Dong Zhu

**Affiliations:** Department of Biology, McMaster University, 1280 Main St. West, Hamilton, Ontario L8S4K1, Canada

## Abstract

TRF1, a duplex telomeric DNA-binding protein, plays an important role in telomere metabolism. We have previously reported that a fraction of endogenous TRF1 can stably exist free of telomere chromatin when it is phosphorylated at T371 by Cdk1; however, the role of this telomere-free (pT371)TRF1 has yet to be fully characterized. Here we show that phosphorylated (pT371)TRF1 is recruited to sites of DNA damage, forming damage-induced foci in response to ionizing radiation (IR), etoposide and camptothecin. We find that IR-induced (pT371)TRF1 foci formation is dependent on the ATM- and Mre11/Rad50/Nbs1-mediated DNA damage response. While loss of functional BRCA1 impairs the formation of IR-induced (pT371)TRF1 foci, depletion of either 53BP1 or Rif1 stimulates IR-induced (pT371)TRF1 foci formation. In addition, we show that TRF1 depletion or the lack of its phosphorylation at T371 impairs DNA end resection and repair of nontelomeric DNA double-strand breaks by homologous recombination. The lack of TRF1 phosphorylation at T371 also hampers the activation of the G2/M checkpoint and sensitizes cells to PARP inhibition, IR and camptothecin. Collectively, these results reveal a novel but important function of phosphorylated (pT371)TRF1 in facilitating DNA double-strand break repair and the maintenance of genome integrity.

## INTRODUCTION

DNA double-strand breaks (DSBs), a lethal form of DNA damage, can promote tumorigenesis if not repaired properly. Sensing of DSBs is mediated by ATM, a PI-3 kinase that transduces the DNA damage signal through phosphorylation of many proteins essential for the activation of the DNA damage checkpoint, cell cycle arrest, DNA repair or apoptosis (1,2). Following the induction of DSBs, γH2AX, resulting from the phosphorylation of histone variant H2AX at serine 139 by ATM (3,4), marks damaged chromatin and directs the recruitment of many DNA damage signaling and DNA repair proteins into repair centers, also known as ‘foci’ (2,5).

Repair of DSBs is mediated by two major repair pathways: nonhomologous end joining (NHEJ) and homologous recombination (HR) (5). NHEJ, error-prone, can ligate two broken ends in the absence of sequence homology, whereas HR, largely error-free, requires sequence homology and is often restricted to the S and G2 phases of the cell cycle during which sister chromatids are present. An error in the choice of the DNA DSB repair pathway can lead to genomic instability. The tumor suppressor proteins 53BP1 and BRCA1 have been shown to play pivotal roles in influencing the fate of the repair of DSBs by either NHEJ or HR (5). While 53BP1 is found to inhibit HR and to promote NHEJ, BRCA1 antagonizes 53BP1 at DSBs, allowing HR to proceed (6–9). BRCA1 is thought to facilitate DNA end resection (6), an early step of HR marked by the generation of RPA-coated single-stranded DNA.

TRF1, a duplex telomeric DNA-binding protein (10), is a component of the six-subunit shelterin complex essential for maintaining telomere length and integrity (11). TRF1 is best known for its role in telomere metabolism (11), but it has also been found to interact with proteins involved in the DNA damage response, such as ATM (12,13) and Mre11/Rad50/Nbs1 (14). Whether and how TRF1 may play a role in the DNA damage response and DNA repair is poorly understood. TRF1 is predominantly found at human telomeres (15); however, a fraction of endogenous TRF1 can also stably exist free of telomere chromatin in the nucleus (16). We have previously reported that TRF1 is phosphorylated at T371 by Cdk1 and that this phosphorylation keeps TRF1 free of telomere chromatin and protects it from proteasome-mediated protein degradation (16). While T371 phosphorylation is upregulated in mitosis to facilitate the separation of sister telomeres (16), a low level of phosphorylated (pT371)TRF1 is also detected in interphase cells (16). However the role of this unbound (pT371)TRF1 in interphase has yet to be characterized.

In this report, using a phospho-specific anti-pT371 antibody, we have shown that telomere-free phosphorylated (pT371)TRF1 forms damage-induced foci in response to ionizing radiation (IR), camptothecin (CPT) and etoposide, indicative of its association with DSBs. We have shown that inhibition of Cdk activity severely impairs the formation of IR-induced (pT371)TRF1 foci, consistent with our previous finding that Cdk1 phosphorylates TRF1 at T371 (**16**). We have demonstrated that an amino acid substitution abrogating TRF1 binding to telomeric DNA stimulates the recruitment of exogenously expressed Myc-tagged TRF1 to sites of DNA damage in a manner dependent on T371 phosphorylation, further supporting the notion that it is telomere-free phosphorylated (pT371)TRF1 that is recruited to sites of DNA damage. We have found that the recruitment of phosphorylated (pT371)TRF1 to sites of DNA damage requires the ATM- and Mre11/Rad50/Nbs1-dependent DNA damage response. While the formation of IR-induced (pT371)TRF1 foci is impaired by loss or depletion of BRCA1, it is stimulated by knockdown of 53BP1 or its downstream effector Rif1 (17–21). Furthermore, we have demonstrated that phosphorylated (pT371)TRF1 not only facilitates DNA end resection and HR, but also activates the G2/M checkpoint and confers cell survival following the induction of DSBs. Taken together, these results have uncovered an important role of phosphorylated (pT371)TRF1 in DNA DSB repair.

## MATERIALS AND METHODS

### Plasmids and antibodies

Expression constructs for shTRF1 and various TRF1 mutant alleles (T371A, T371D, R425V) have been previously described (16). Wild-type Nbs1 was cloned into pLPC retroviral vector with a Myc epitope tag replacing the start codon. Nbs1 deletion constructs were generated through polymerase chain reaction using wild-type Nbs1 as a template. The sequence of primers for cloning Nbs1 deletion alleles will be made available upon request. The annealed oligonucleotides encoding small interfering RNAs previously described for ATM (22), BRCA1 (23) and 53BP1 (24) were ligated into the pRetroSuper vector, giving rise to knockdown expression constructs used in this study. Small interference RNA against Rif1 was a generous gift from Daniel Durocher, Samuel Lunenfeld Research Institute.

Phospho-specific anti-pT371-TRF1 has been previously described (16). Other antibodies used include TRF1 (a gift from Titia de Lange, Rockefeller University); Nbs1 (kindly provided by John Petrini, Memorial Sloan-Kettering Cancer Center); Rif1 (kindly provided by Daniel Durocher, Samuel Lunenfeld Research Institute); BRCA1 (MS110, Abcam); RPA32 (9H8, Abcam); Chk1-pS317 (Bethyl); RPA32-pS4/pS8 (Bethyl); ATM-pS1981 (Cell Signaling); H3-pS10 (Cell Signaling); Chk1 (FL-476, Santa Cruz); γ-H2AX (Milipore); 53BP1 (BD Biosciences); ATM (Novus) and γ-tubulin (GTU88, Sigma).

### Cell culture and treatments

Cells were grown in Dulbecco's modified Eagle's medium with 10% fetal bovine serum supplemented with non-essential amino acids, l-glutamine, 100 U/ml penicillin and 0.1 mg/ml streptomycin. HT1080, MCF7 and HCC1937 are cell lines from ATCC. GM637, GM16666 (AT-deficient) and GM16667 (ATM-complemented) are cell lines from Coriell. Pheonix cells, NBS-ILB1, HeLaI.2.11 and HeLaII were gifts from Titia de Lange, Rockefeller University. HeLaI.2.11 and HeLaII are two sublines of HeLa cells of different telomere length (25). Retroviral gene delivery was carried out as described (16,26).

To inhibit Cdk1 activity, 20 µM Roscovitine (Sigma) or 2 µM CGP74541A (Sigma) was added to cells 4 h before IR treatment. For inhibition of ATM or Mre11, cells were treated with either KU55933 (20 µM, Sigma) or Mirin (50 µM, Sigma) for 1 h before IR treatment. For damage induction, cells were treated with etoposide (1 µM, Sigma) or CPT (1 µM, Sigma) for 1 h. IR was delivered from a Cs-137 source at McMaster University (Gammacell 1000).

### Differential salt extraction of chromatin and immunoblotting

Protein extracts, differential salt extraction of chromatin and immunoblotting were performed as described (16,26). For IR-treated cells, protein extracts were in general prepared 8 h before IR except where specified.

### Immunofluorescence and fluorescence *in situ* hybridization

Immunofluorescence (IF) was performed as described (26,27). IF-fluorescence *in situ* hybridization (FISH) analyses were conducted as described (28). For IR-treated cells, cells were fixed in general 8 h after IR except where specified. For each treatment or time point, 500–1000 cells were scored in blind for a single experiment and a minimum of three independent experiments were performed.

### Analysis of metaphase chromosome aberration

Analysis of chromosome aberration was carried out as described (29) with modifications. Cells were treated with 1 Gy IR and allowed to recover in the incubator for 1 h before the addition of 100 ng/ml nocodazole. Following a 4 h incubation in the presence of nocodazole, cells were collected by shake-off, incubated for 7 min at 37°C in 75 mM KCl and then fixed in freshly made methanol/acetic acid (3:1). On the following day, cells were dropped onto slides and air-dried overnight at RT in a chemical hood. The dried slides with chromosome spreads were subsequently processed according to the FISH protocol as described (28,30) except that the steps of the denaturation and the incubation with telomeric DNA-containing peptide nucleic acid (PNA) probe were omitted. All cell images were recorded on a Zeiss Axioplan 2 microscope with a Hammamatsu C4742-95 camera and processed in Open Lab.

### DNA recombinational repair assays

Three reporter plasmids pDR-GFP, pSA-GFP and pEGFP-Pem1-Ad2, generously provided by Eric Hendrickson, University of Minnesota, were used to assess HR, single-strand annealing and NHEJ, respectively, as described (31). In brief, lipofectamine LTX plus reagent (Invitrogen) was used to transfect cells with *I-Sce*I-expressing plasmid, pCherry and pDR-GFP, pSA-GFP or pEGFP-Pem1-Ad2 in a ratio of 1:0.5:1 according to manufacture’s instructions. Forty-eight hours after transfection, cells were harvested and subjected to fluorescence-activated flow cytometry (FACS) analysis. The number of cells positive for both GFP and pCherry was normalized to the total number of pCherry-positive cells, giving rise to percentage of GFP-positive cells. For FACS analysis, cells were harvested, washed in 1× phosphate buffered saline (PBS) and fixed in PBS-buffered 4% paraformaldehyde. FACS analysis was performed using a Becton-Dickinson LSRII located at the SickKids-UHN flow cytometry facility, Toronto, ON, Canada.

### Clonogenic survival and G2/M checkpoint assays

For clonogenic survival assays, 4 h before CPT treatment, HT1080 and TRF1-depeleted HeLaII cells stably expressing various TRF1 alleles were seeded in duplicates at 120 cells (0–50 nM CPT) or 720 cells (100 nM CPT) per 6-cm plate. After 1 h of CPT treatment, the drug was washed off with PBS and fresh growth medium was added. For IR treatment, cells were counted, irradiated and seeded in duplicates at 120 cells (0–3 Gy) or at 720 cells (5 Gy) per 6-cm plate, followed by replacement with fresh media after 24 h incubation. For poly (ADP-ribose) polymerase (PARP) inhibitor treatment, 24 h after seeding, cells were treated with Olaparib (Selleck Chemicals) and allowed to grow in the presence of Olaparib for the entirety of the experiments. Ten days later, colonies were fixed and stained at RT for 10 min with a solution containing 50% methanol, 7% acetic acid and 0.1% Coomassie blue. Colonies consisting of >32 cells were scored.

For the G2/M checkpoint assay, cells seeded on coverslips were treated with 12 Gy IR and allowed to recover in the incubator. Following 1 h incubation, cells were gently washed with PBS, fixed with paraformaldehyde and then processed for IF with anti-H3-pS10 antibody.

### Statistical analysis

A Student’s two-tailed *t*-test was used to derive all *P*-values.

## RESULTS

### Phosphorylated (pT371)TRF1 is recruited to sites of DNA DSBs

**Figure 1.**
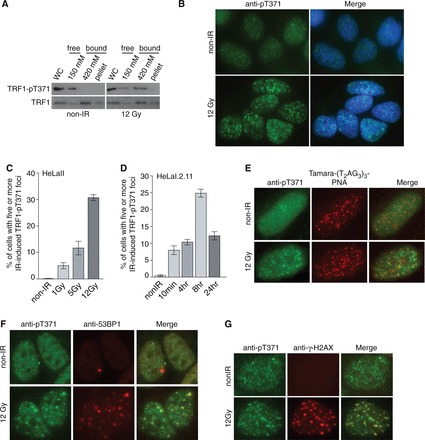
Phosphorylated (pT371)TRF1 forms IR-induced damage foci. (**A**) Analysis of differential salt extraction of chromatin of mock- or 12 Gy IR-treated HeLaII cells. Western analysis was performed with anti-pT371 or anti-TRF1 antibody. WC: whole cell lysate. (**B**) Indirect IF with anti-pT371 antibody on HeLaII cells with or without 12 Gy IR treatment. Cell nuclei were stained with DAPI in blue. (**C**) Quantification of the percentage of HeLaII cells with five or more IR-induced (pT371)TRF1 foci. HeLaII cells were treated with a varying dose of IR as indicated. Standard deviations from three independent experiments are indicated. (**D**) Quantification of the percentage of HeLaI.2.11 cells with five or more IR-induced (pT371)TRF1 foci. HeLaI.2.11 were treated with 12 Gy IR and then fixed at various time points as indicated. Standard deviations from three independent experiments are indicated. (**E**) IF-FISH with anti-pT371 antibody (green) in conjunction with Tamara-conjugated-(T_2_AG_3_)_3_ PNA probe (red). HeLaII cells were mock treated or treated with 12 Gy IR. (**F**) Indirect IF with anti-pT371 in conjunction with anti-53BP1 antibody on HeLaII cells with or without IR treatment. Cell nuclei were stained with DAPI in blue. (**G**) Indirect IF with anti-pT371 in conjunction with anti-γH2AX antibodies on HeLaII cells treated with or without IR treatment. Cell nuclei were stained with DAPI in blue.

Previously we have reported that Cdk1 phosphorylates T371 of TRF1 and that this phosphorylation prevents TRF1 from associating with telomeric DNA both *in vivo* and *in vitro* (16). To investigate the biological function of TRF1 phosphorylation at T371 in interphase, we treated HeLaII cells with IR, followed by analysis of differential salt extraction of chromatin with our previously characterized phospho-specific anti-pT371 antibody (16). We found that treatment with 12 Gy IR had little effect on the chromatin association of total TRF1 (Figure 1A), which was found mainly in the chromatin-bound fraction (Figure 1A), consistent with previous findings (16). In contrast, we observed a pronounced shift of phosphorylated (pT371)TRF1 from the chromatin-free fraction to the chromatin-bound fraction following 12 Gy radiation (Figure 1A), suggesting that phosphorylated (pT371)TRF1 may be associated with damaged chromatin.

To further investigate the damage-induced chromatin association of phosphorylated (pT371)TRF1, we performed indirect IF with phospho-specific anti-pT371 antibody and found that phosphorylated (pT371)TRF1 formed IR-induced foci in a number of human cell lines including HeLa, GM637 and hTERT-BJ cells (Figure 1B and Supplementary Figure S1A and B), suggesting that IR-induced (pT371)TRF1 foci formation is not cell type specific. The formation of IR-induced (pT371)TRF1 foci was dose-dependent (Figure 1C) and peaked 8 h after IR (Figure 1D).

Analysis of IF-FISH with anti-pT371 antibody in conjunction with telomeric DNA-containing PNA probe revealed little overlap between IR-induced (pT371)TRF1 foci and human telomeres (Figure 1E), suggesting that damage-induced chromatin association of (pT371)TRF1 is independent of the presence of telomeric DNA. On the other hand, we found that IR-induced (pT371)TRF1 foci merged well with IR-induced foci of 53BP1 (Figure 1F) or γ-H2AX (Figure 1G), both of which are known to accumulate at sites of DSBs (2). Aside from IR, we also observed damage-induced phosphorylated (pT371)TRF1 foci in cells treated with DSB-inducing agents such as CPT or etoposide (Supplementary Figure S1C and D). Collectively, these results suggest that phosphorylated (pT371)TRF1 is recruited to sites of DSBs.

**Figure 2.**
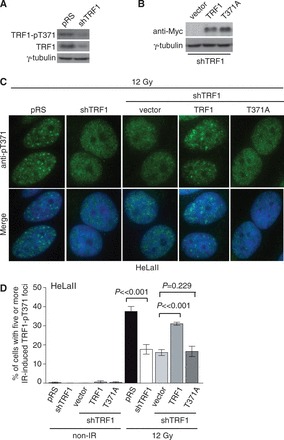
Depletion of TRF1 impairs the formation of IR-induced (pT371)TRF1 foci. (**A**) Western analysis of TRF1 depletion in HeLaII cells. Immunoblotting was performed with anti-pT371 or anti-TRF1 antibody. The γ-tubulin blot was used as a loading control in this and the following experiments. (**B**) Western analysis of shTRF1-resistant Myc-tagged wild-type or mutant TRF1 proteins in TRF1-depleted HeLaII cells. (**C**) Indirect IF with anti-pT371 antibody. HeLaII cells expressing various constructs as indicated were treated with 12 Gy IR. Cell nuclei were stained with DAPI in blue. (**D**) Quantification of the percentage of cells containing five or more IR-induced (pT371)TRF1 foci from (C). Standard deviations from six independent experiments are indicated.

To address the possiblity that our phospho-specific anti-pT371 antibody might cross react with other DNA damage response factors, we depleted endogenous TRF1 in HeLaII cells (Figure 2A) and then complemented TRF1-depleted HeLaII cells with Myc-tagged shTRF1-resistant wild-type TRF1 or TRF1 carrying a nonphosphorylatable T371A mutation (Figure 2B). We found that TRF1 depletion significantly impaired the formation of IR-induced (pT371)TRF1 foci (Figure 2C and D). While the introduction of Myc-tagged wild-type TRF1 was able to rescue the formation of IR-induced (pT371)TRF1 foci in TRF1-depleted HeLaII cells (Figure 2C and D), no rescue in the formation of these foci was observed when TRF1-depleted HeLaII cells were complemented with Myc-tagged TRF1 carrying the nonphosphorylatable T371A mutation (Figure 2C and D). The level of expression of Myc-tagged TRF1-T371A was indistinguishable from that of Myc-tagged wild-type TRF1 (Figure 2B). These results argue against the possibility that the observed IR-induced (pT371)TRF1 foci are due to a cross-reactivity of the phospho-specific anti-pT371 antibody with other DNA damage response proteins.

**Figure 3.**
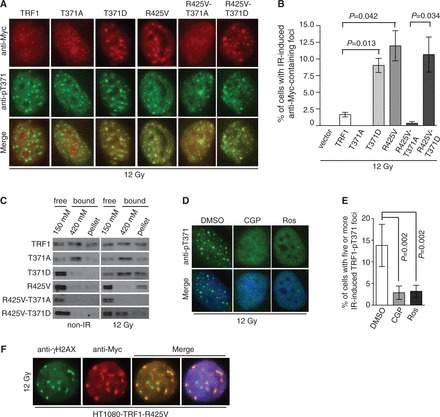
Phosphorylation of T371 by Cdk1 directs telomere-free TRF1 to sites of DSBs. (**A**) Indirect IF with mouse anti-Myc antibody (red) in conjunction with rabbit anti-pT371 antibody (green). HT1080 cells stably expressing various Myc-tagged TRF1 alleles as indicated were treated with 12 Gy IR. (**B**) Quantification of the percentage of cells containing five or more IR-induced anti-Myc-containing foci from (A). Standard deviations from three independent experiments are indicated. (**C**) Analysis of differential salt extraction of chromatin. HT1080 cells stably expressing various Myc-tagged TRF1 alleles as indicated were mock treated or treated with 12 Gy IR. Immunoblotting was performed with anti-Myc antibody. (**D**) Indirect IF with anti-pT371 antibody. HeLaII cells pretreated with DMSO, CGP74514A (CGP) or Roscovitine (Ros) were subjected to IR and fixed 8 h after IR. Cell nuclei were stained with DAPI in blue. (**E**) Quantification of the percentage of cells with five or more IR-induced (pT371)TRF1 foci from (D). Standard deviations from at least three independent experiments are indicated. (**F**) Indirect IF with rabbit anti-γH2AX antibody in conjunction with mouse anti-Myc antibody. HT1080 cells overexpressing Myc-tagged TRF1 carrying the R425V mutation were treated with 12 Gy IR and fixed 8 h after IR.

To further demonstrate the association of phosphorylated (pT371)TRF1 with damaged DNA, we examined the ability of exogenously expressed Myc-tagged wild-type TRF1, TRF1 carrying a nonphosphorylatable T371A mutation or TRF1 with a phosphomimic T371D mutation to form IR-induced foci. Analysis of indirect IF with anti-Myc antibody revealed that no IR-induced anti-Myc-containing foci was observed for Myc-TRF1-T371A-expressing HT1080 cells, whereas at least 9% of HT1080 cells overexpressing Myc-TRF1-T371D exhibited IR-induced anti-Myc-containing foci (Figure 3A and B). Less than 2% of HT1080 cells overexpressing Myc-tagged wild-type TRF1 exhibited IR-induced anti-Myc containing foci (Figure 3B), indicating that Myc-tagged TRF1 was also able to form IR-induced foci albeit at a much lower frequency than Myc-TRF1-T371D. Previously we have reported that 1–5% of endogenous TRF1 in cells is phosphorylated at T371 (16). The low abundance of T371 phosphorylation may in part account for the observed low frequency of IR-induced Myc-TRF1-containing foci. Futhermore, analysis of differential salt extraction of chromatin revealed that while Myc-tagged TRF1-T371D was predominantly found in the chromatin-free fraction of untreated cells (Figure 3C), consistent with our previous finding (16), it became largely associated with chromatin following IR (Figure 3C). Taken together, these results suggest that phosphorylation at T371 directs TRF1 to sites of DNA damage. In support of this notion, we also found that the treatment of HeLaII cells with roscovitine or a second selective Cdk1 inhibitor CGP74541A significantly impaired the formation of IR-induced (pT371)TRF1 foci (Figure 3D and E), consistent with our previous finding that T371 is a target of Cdk1 (16). These results suggest that phosphorylation of T371 by Cdk1 is required for the association of TRF1 with damaged DNA.

### Being telomere free is necessary for (pT371)TRF1 recruitment to sites of DNA damage

TRF1 is a duplex telomeric DNA-binding protein (10) and therefore we decided to investigate whether binding to telomeric DNA might affect TRF1 association with DSBs. Previously we and others have reported that TRF1 carrying a single amino acid substitution of R425V in the Myb-like DNA binding domain is defective in binding to telomeric DNA both *in vivo* and *in vitro* (16,32). We therefore generated human fibroblastoma HT1080 cell lines stably overexpressing Myc-tagged wild-type TRF1 or Myc-tagged TRF1-R425V. Analysis of IF-FISH revealed that Myc-tagged TRF1-R425 failed to exhibit any punctate telomere staining (Supplementary Figure S2), in line with previous reports that it is defective in binding to telomeric DNA (16,32). We found that Myc-tagged TRF1-R425V was able to form IR-induced damaged foci that colocalized with not only endogenous (pT371)TRF1 (Figure 3A) but also γH2AX (Figure 3F). In addition, we observed a 6-fold induction of IR-induced anti-Myc containing foci in Myc-TRF1-R425V-expressing cells compared with Myc-TRF1-expressing cells (Figure 3A and B). Furthermore, analysis of differential salt extraction of chromatin revealed that Myc-TRF1-R425V was predominantly found in the chromatin-free fraction in untreated cells (Figure 3C), consistent with previous findings that it is defective in binding to telomeric DNA (16,32); however, a substantial amount of it became associated with chromatin following IR treatment (Figure 3C). Introduction of a nonphosphorylatable T371A mutation into Myc-TRF1-R425V completely abrogated not only its ability to form IR-induced foci (Figure 3A and B) but also its IR-induced chromatin association (Figure 3C). On the other hand, a phosphomimic T371D mutation had little effect on the ability of Myc-TRF1-R425V to form IR-induced foci (Figure 3A and B), nor did it affect its IR-induced chromatin association (Figure 3C). Taken together, these results suggest that phosphorylation of T371 directs telomere-free TRF1 to sites of DNA damage.

### TRF1 phosphorylation at T371 facilitates DNA end resection and HR

**Figure 4.**
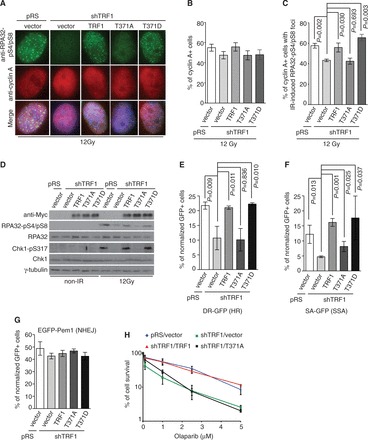
TRF1 phosphorylation at T371 facilitates DNA end resection and HR. (**A**) Indirect IF with anti-RPA32-pS4/pS8 in conjunction with anti-cyclin A antibody. TRF1-depleted HeLaII cells stably expressing the vector alone or various TRF1 alleles as indicated were treated with 12 Gy and fixed 8 h later. Cell nuclei were stained with DAPI in blue. (**B**) Quantification of cyclin A-positive cells in TRF1-depleted cells expressing various alleles as indicated. Standard deviations from six independent experiments are indicated. (**C**) Quantification of the percentage of cyclin A-positive cells with five or more IR-induced RPA32-pS4/pS8 foci from (A). Standard deviations from six independent experiments are indicated. (**D**) Western analysis of mock- or IR-treated TRF1-depleted HeLaII cells stably expressing the vector alone or various TRF1 alleles as indicated. Immunoblotting was carried out with anti-RPA32-pS4/pS8, anti-RPA32, anti-Chk1-pS317, anti-Chk1 or anti-γ-tubulin. (**E**) HR-mediated repair of I-SceI-induced DSBs. TRF1-depleted HeLaII cells expressing various TRF1 alleles as indicated were cotransfected with pDR-GFP, pCherry and I-SceI expression constructs. The number of cells positive for both GFP and pCherry was normalized to the total number of pCherry-positive cells, giving rise to the percentage of normalized GFP-positive cells. Standard deviations from four independent experiments are indicated. (**F**) SSA-mediated repair of I-SceI-induced DSBs. TRF1-depleted HeLaII cells expressing various TRF1 alleles as indicated were cotransfected with pSA-GFP, pCherry and I-SceI expression constructs. The quantification was done as described in (E). Standard deviations from three independent experiments are indicated. (**G**) NHEJ-mediated repair of I-SceI-induced DSBs. TRF1-depleted HeLaII cells expressing various TRF1 alleles as indicated were cotransfected with pEGFP-Pem1-Ad2, pCherry and I-SceI expression constructs. The quantification was done as described in (E). Standard deviations from three independent experiments are indicated. (**H**) Clonogenic survival assays of Olaparib-treated HeLaII cells stably expressing various alleles as indicated. Standard deviations from three independent experiments are indicated.

Previous studies have shown that HR is a slow repair process, which can take ≥7 h to complete, whereas NHEJ can be completed in ∼30 min following the induction of DSBs (33–38). Our earlier finding that IR-induced (pT371)TRF1 foci formation peaked 8 h after IR suggests that (pT371)TRF1 may be involved in HR-mediated DNA repair. To investigate this possibility, we examined the formation of IR-induced RPA32-pS4/pS8 foci, a readout commonly used for DNA end resection (39,40), in TRF1-depleted HeLaII cells expressing wild-type TRF1 or TRF1 carrying either a nonphosphorylatable or a phosphomimic mutation at T371. We found that depletion of TRF1 or overexpression of various TRF1 alleles did not significantly alter the percentage of cells in S and G2 phases as measured by cyclin A staining (Figure 4A and B). Analysis of dual indirect immunoflorescence with anti-RPA32-pS4/pS8 antibody in conjunction with anti-cyclin A antibody revealed that knockdown of TRF1 led to a significant reduction in the number of cyclin A-positive cells exhibiting IR-induced RPA32-pS4/S8 foci (Figure 4A and C). IR-induced RPA foci in TRF1-depleted cells were rescued by overexpression of shTRF1-resistant wild-type TRF1 or TRF1 carrying a T371D mutation (Figure 4A and C). On the other hand, no rescue for IR-induced RPA32-pS4/S8 foci was observed in TRF1-depleted cells overexpressing TRF1-T371A (Figure 4A and C). Western analysis revealed that depletion of TRF1 resulted in a loss of IR-induced phosphorylation of S4/S8 of RPA32 (Figure 4D). This loss was suppressed by shTRF1-resistant wild-type TRF1 or TRF1 carrying the phosphomimic T371D mutation (Figure 4D). In contrast, TRF1 carrying the T371A mutation failed to restore S4/S8 phosphorylation of RPA32 in TRF1-depleted cells (Figure 4D). Taken together, these results suggest that TRF1 phosphorylation at T371 plays an important role in facilitating DNA end resection.

To further investigate the role of (pT371)TRF1 in HR, we used three well-established reporter plasmids; pDR-GFP (41), pSA-GFP (42) and pEGFP-Pem1-Ad2 (43). These reporter plasmids contain GFP disrupted by the insertion of an I-SceI site. HR-mediated repair of I-SceI-induced DSBs restores GFP expression in pDR-GFP, whereas repair of I-SceI-induced DSBs by single-strand annealing (SSA), a subpathway of HR, restores GFP expression in pSA-GFP. On the other hand, NHEJ-mediated repair of I-SceI-induced DSBs restores GFP expression in pEGFP-Pem1-Ad2. We found that depletion of TRF1 in HeLaII cells impaired HR- or SSA-mediated restoration of GFP expression (Figure 4E and F), whereas it had little effect on NHEJ-mediated restoration of GFP expression (Figure 4G). We observed that when introduced into TRF1-depleted cells, shTRF1-resistant wild-type TRF1 or TRF1 carrying a phosphomimic T371D mutation was able to rescue HR- or SSA-mediated restoration of GFP expression, whereas shTRF1-resistant TRF1 carrying a nonphosphorylatable T371A mutation failed to do so (Figure 4E and F). Overexpression of wild-type TRF1 or TRF1 carrying either a T371A or a T371D mutation had little effect on NHEJ-mediated restoration of GFP (Figure 4G). Furthermore, we found that depletion of TRF1 sensitized HeLaII cells to Olaparib, a PARP inhibitor known to be toxic to HR-deficient cells (17) (Figure 4H). This sensitivity was suppressed when TRF1-depleted HeLaII cells were complemented with shTRF1-resistant wild-type TRF1 (Figure 4H). In constrast, overexpression of shTRF1-resistant TRF1 carrying a mutation of T371A was not able to reverse the sensitivity of TRF1-depleted cells to Olaparib (Figure 4H). Taken together, these results reveal that TRF1 phosphorylation at T371 promotes the repair of DSBs by HR.

### TRF1 faciliates the activation of the G2/M checkpoint and the maintenance of genome integrity

**Figure 5.**
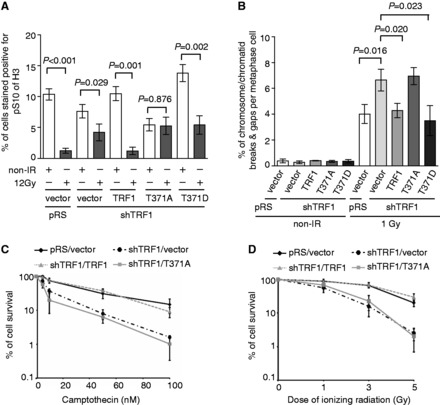
The lack of TRF1 phosphorylation at T371 impairs the activation of the G2/M checkpoint and hampers the maintenance of genome integrity. (**A**) Quantification of the percentage of cells stained positive for H3-pS10. For each cell line, 3000 cells were scored in blind. Standard deviations from four independent experiments are indicated. (**B**) Quantification of the percentage of chromosome/chromatid breaks and gaps per metaphase cell. TRF1-depleted HeLaII cells stably expressing the vector alone or various TRF1 alleles as indicated were mock treated or treated with 1 Gy IR. For each cell line, 60 metaphase cells were scored in blind. Standard deviations from three independent experiments are indicated. (**C** and **D**) Clonogenic survival assays following various doses of CPT (C) or IR (D). The colony forming assays were performed with TRF1-depleted HeLaII cells stably expressing the vector alone or various TRF1 alleles as indicated. HeLaII cells expressing both pRS and pWZL vectors were also included as a control. Standard deviations from six independent experiments are indicated.

We found that depletion of TRF1 abrogated ATR-dependent phosphorylation of S317 of Chk1 following IR (Figure 4D). Introduction of wild-type TRF1 or TRF1 carrying a phosphomimic T371D mutation was able to rescue IR-induced S317 phosphorylation of Chk1 in TRF1-depleted cells, whereas no rescue of IR-induced S317 phosphorylation was observed in TRF1-depleted cells complemented with TRF1 carrying a nonphosphorylatable T371A mutation (Figure 4D). In addition, analysis of the mitotic index following IR revealed that TRF1-depleted HeLaII cells overexpressing TRF1 carrying a nonphosphorylatable T371A mutation failed to undergo a G2/M arrest, whereas the IR-induced G2/M arrest was observed in TRF1-depleted cells overexpressing wild-type TRF1 or TRF1 carrying a phosphomimic T371D mutation (Figure 5A). Overexpression of TRF1-T371A was also observed to mitigate the IR-induced G2/M arrest in HT1080 cells (Supplementary Figure S3A). These results suggest that TRF1 phosphorylation at T371 is important for facilitating the activation of the ATR-Chk1-mediated G2/M checkpoint.

Analysis of metaphase chromosome spreads revealed that depletion of TRF1 promoted an accumulation of IR-induced chromosome/chromatid breaks and gaps (Figure 5B), in line with the notion that TRF1 facilitates DSB repair by HR. Overexpression of shTRF1-resistant wild-type TRF1 or TRF1 carrying a phosphomimic T371D mutation was able to suppress the level of IR-induced chromosome/chromatid breaks and gaps in TRF1-depleted cells (Figure 5B), which was not reduced when TRF1-depleted cells were complemented with TRF1 carrying the T371A mutation (Figure 5B). Furthermore, we found that depletion of TRF1 increased the sensitivity of cells to IR and CPT (Figure 5C and D), indicative of its role in promoting cell survival following the induction of DNA DSBs. The hypersensitivity of TRF1-depleted cells to IR or CPT was rescued by wild-type TRF1 but not by TRF1 carrying a T371A mutation (Figure 5C and D). The inability of TRF1 carrying a T371A mutation to facilitate cell survival was also observed in HT1080 cells treated with IR or CPT (Supplementary Figure S3B and C). Expression of TRF1-T371A was comparable with that of wild-type TRF1 or TRF1-T371D (Supplementary Figure S3D). Together, these results demonstrate an important function of phosphorylated (pT371)TRF1 in maintaining genome integrity and promoting cell survival.

### (pT371)TRF1 recruitment to sites of DNA DSBs requires the ATM- and Mre11/Rad50/Nbs1-dependent DNA damage response

**Figure 6.**
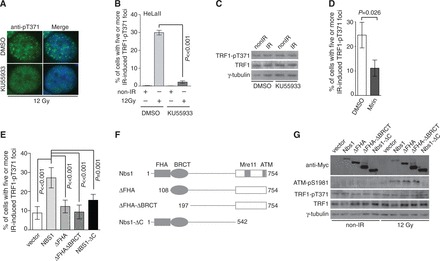
The formation of IR-induced (pT371)TRF1 foci is dependent on the ATM- and Mre11/Rad50/Nbs1-mediated DNA damage response. (**A**) Indirect IF with anti-pT371 antibody. Before IR, HeLaII cells were treated with DMSO or KU55933. Cell nuclei were stained with DAPI in blue. (**B**) Quantification of the percentage of cells with five or more IR-induced (pT371)TRF1 foci from (A). Standard deviations from three independent experiments are indicated. (**C**) Western analysis. HeLaII cells treated with DMSO or KU55933 were subjected to mock treatment or 12 Gy IR. Immunoblotting was carried out with anti-pT371, anti-TRF1 and anti-γ-tubulin antibody. (**D**) Quantification of the percentage of DMSO- or Mirin-treated HeLaII cells with five or more IR-induced (pT371)TRF1 foci. Standard deviations from three independent experiments are shown. (**E**) Quantification of the percentage of cells with five or more IR-induced (pT371)TRF1 foci. Standard deviations from seven independent experiments are indicated. Nbs1-deficient cells (NBS-ILB1) complemented with the vector alone or various Myc-tagged Nbs1 alleles as indicated were mock treated or treated with 12 Gy IR. (**F**) Schematic diagram of Nbs1 alleles. (**G**) Western analysis. Nbs1-deficient cells (NBS-ILB1) complemented with the vector alone or various Myc-tagged Nbs1 alleles as indicated were mock treated or treated with 12 Gy IR. Immunoblotting was done with anti-Myc, anti-ATM-pS1981, anti-pT371, anti-TRF1 or anti-γ-tubulin antibody.

To investigate the mechanism by which phosphorylated (pT371)TRF1 is recruited to sites of DSBs to facilitate HR, we examined the relationship between the DNA damage response pathway and phosphorylated (pT371)TRF1. Upon the induction of DSBs, a number of DNA damage response factors are recruited to sites of DNA damage including ATM, γH2AX, Mre11/Rad50/Nbs1, BRCA1 and 53BP1. Recruitment of these proteins to sites of DSBs following IR was not affected by depletion of TRF1 or overexpression of TRF1 carrying a nonphosphorylatable T371A mutation (M. McKerlie and X.-D. Zhu, unpublished data), suggesting that T371 phosphorylation is not involved in sensing DSBs. On the other hand, we found that treatment of cells with KU55933, a specific inhibitor of ATM, completely abrogated the ability of (pT371)TRF1 to form IR-induced foci (Figure 6A and B). The defect in the ability of (pT371)TRF1 to form IR-induced foci was also observed in HeLaII cells knocked down for ATM (Supplementary Figure 4A and B) as well as in cells derived from an AT patient (Supplementary Figure S4D and E). Inhibition, knockdown or loss of ATM had little effect on the level of T371 phosphorylation (Figure 6C and Supplementary Figure S4C and F). These results suggest that ATM is required for the recruitment of (pT371)TRF1 to sites of DSBs.

It has been reported that the Mre11/Rad50/Nbs1 complex is required for ATM activation following the induction of DSBs (44,45). Therefore we also examined the role of the Mre11/Rad50/Nbs1 complex in recruiting (pT371)TRF1 to DSBs. We found that treatment of HeLa cells with Mirin, a specific inhibitor of Mre11 (46), impaired IR-induced ATM-pS1981 foci formation (Supplementary Figure S5A and B), in agreement with the previous finding that Mirin prevents ATM autophosphorylation on S1981 (46). Analysis of indirect IF with anti-pT371 antibody revealed that treatment with Mirin also significantly impaired the ability of (pT371)TRF1 to form IR-induced foci (Figure 6D and Supplementary Figure S5C), although it had little effect on the level of T371 phosphorylation (Supplementary Figure S5D). In addition, we found that the complementation of wild-type Nbs1 into Nbs1-deficient cells led to at least a 3-fold increase (*P* < 0.001) in the formation of IR-induced (pT371)TRF1 foci (Figure 6E and Supplementary Figure S6), suggesting that loss of Nbs1 impairs IR-induced (pT371)TRF1 foci formation.

The C-terminal 220 amino acids of Nbs1 have also been reported to bind TRF1 in a yeast two-hybrid assay (14). Nbs1 contains forkhead associated (FHA) and BRCT-repeat domains (Figure 6F), both of which are known to bind phosphorylated threonine/serine. These findings prompted us to examine whether a particular domain(s) of Nbs1 might mediate the recruitment of (pT371)TRF1 to sites of DSBs. To address this question, we generated Myc-tagged Nbs1 lacking the FHA domain alone (Myc-Nbs1-ΔFHA), both FHA and BRCT domains (Myc-Nbs1-ΔFHA-ΔBRCT) or the C-terminal TRF1-interacting domain (Myc-Nbs1-ΔC) (Figure 6F). These alleles were examined for their ability to rescue IR-induced (pT371)TRF1 foci in Nbs1-deficient cells. We found that while full-length Nbs1 rescued the formation of IR-induced (pT371)TRF1 foci in Nbs1-deficient cells (Figure 6E and Supplementary Figure S6), Nbs1 lacking either the N-terminal FHA domain (Myc-Nbs1-ΔFHA) or the C-terminal domain (Myc-Nbs1-ΔC) failed to rescue IR-induced (pT371)TRF1 foci in Nbs1-deficient cells (Figure 6E and Supplementary Figure S6). The inability of Myc-Nbs1-ΔFHA and Myc-Nbs1-ΔC to rescue IR-induced (pT371)TRF1 foci was unlikely due to a lack of their expression (Figure 6G). Deletion of the C-terminal domain of Nbs1 also failed to activate ATM as evidenced by the lack of IR-induced ATM phosphorylation at S1981 (Figure 6G). Expression of full-length or various Nbs1 deletion alleles had little effect on the level of TRF1 phosphorylation at T371 (Figure 6G). Together, these results suggest that the ATM- and Mre11/Rad50/Nbs1-mediated DNA damage response is required for (pT371)TRF1 recruitment to sites of DSBs. These results further imply that multiple domains of Nbs1 may mediate the recruitment of (pT371)TRF1 to sites of DNA damage.

### BRCA1 and 53BP1 play opposing roles in recruiting (pT371)TRF1 to DSBs

**Figure 7.**
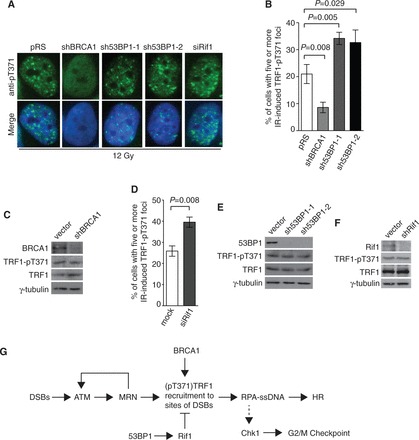
The formation of IR-induced (pT371)TRF1 foci requires BRCA1 but is inhibited by 53BP1 and Rif1. (**A**) Indirect IF with anti-pT371 antibody on 12 Gy IR-treated HeLaII cells depleted for BRCA1 or 53BP1. Knockdown of 53BP1 was performed with two independent shRNA (sh53BP1-1 and sh53BP1-2). Cell nuclei were stained with DAPI in blue. (**B**) Quantification of the percentage of cells with five or more IR-induced (pT371)TRF1 foci in HeLaII cells depleted for BRCA1 or 53BP1 from (**A**). Standard deviations from three independent experiments are indicated. (**C**) Western analysis of HeLaII cell extracts with or without depletion of BRCA1. Immunoblotting was performed with anti-BRCA1, anti-pT371, anti-TRF1 or anti-γ-tubulin antibody. (**D**) Quantification of the percentage of cells with five or more IR-induced (pT371)TRF1 foci in HeLaII cells knocked down for Rif1. Standard deviations from three independent experiments are indicated. (**E**) Western analysis of HeLaII cell extracts with or without depletion of 53BP1. Immunoblotting was performed with anti-53BP1, anti-pT371, anti-TRF1 or anti-γ-tubulin antibody. (**F**) Western analysis of HeLaII cell extracts with or without depletion of Rif1. Immunoblotting was performed with anti-Rif1, anti-pT371, anti-TRF1 or anti-γ-tubulin antibody. (**G**) Model for the role of (pT371)TRF1 in facilitating HR and checkpoint activation. See the text for more information.

BRCA1 is a tumor suppressor protein that facilitates repair of DSBs by HR and its action is antagonized by 53BP1 (6–9). Therefore, we decided to examine the role of both BRCA1 and 53BP1 in the formation of IR-induced (pT371)TRF1 foci. Analysis of indirect IF with anti-pT371 antibody revealed that depletion of BRCA1 impaired the formation of IR-induced (pT371)TRF1 foci in HeLaII cells (Figure 7A and B). The defect of (pT371)TRF1 in forming IR-induced foci was also detected in the breast cancer HCC1937 cells expressing truncated BRCA1 lacking its C-terminal BRCT domain (Supplementary Figure S7). BRCA1 knockdown did not affect the level of T371 phosphorylation (Figure 7C). These results suggest that BRCA1 is required for (pT371)TRF1 recruitment to sites of DSBs.

We found that depletion of 53BP1 with either sh53BP1-1 or sh53BP1-2, two independent shRNA against 53BP1 (24), stimulated the formation of IR-induced (pT371)TRF1 foci (Figure 7A and B) and that this stimulation was also observed in cells knocked down for Rif1, an effector of 53BP1 (17,19,20) (Figure 7D). Knockdown of either 53BP1 or Rif1 had little effect on the level of TRF1 or (pT371)TRF1 (Figure 7E and F). These results suggest that 53BP1 and Rif1 act as antagonists of (pT371)TRF1 recruitment to DSBs.

## DISCUSSION

Many DNA repair proteins are found to be associated with human telomeres and participate in the maintenance of telomere length and integrity. However, whether the process of DNA repair may involve telomere binding proteins has been poorly understood. In this report, we have uncovered that TRF1 phosphorylated at T371 is recruited to sites of DNA damage and that this recruitment is inhibited by 53BP1 and Rif1 but it is dependent on BRCA1 as well as the ATM- and MRN-mediated DNA damage response (Figure 7G). We have demonstrated that phosphorylated (pT371)TRF1 facilitates DNA end resection and HR. Our data presented here reveal a novel and important function of phosphorylated (pT371)TRF1 in facilitating DNA DSB repair and the maintenance of genome integrity.

Our finding that a nonphosphorylatable T371A mutation of TRF1 fails to rescue HR- and SSA-mediated repair of I-SceI-induced DSBs in both pDR-GFP and SSA-GFP reporter constructs demonstrates that phosphorylated (pT371)TRF1 is important for facilitating HR-mediated repair of nontelomeric DNA DSBs. This feature of TRF1 appears to be shared with its related proteins TRF2 and Tbf1. TRF2, another shelterin protein similar to TRF1 that also binds sequence-specifically to duplex telomeric DNA (47,48), has been reported to be recruited to sites of laser-induced DNA damage (49,50) and to promote homologous recombinational repair of nontelomeric DNA DSBs (51). Both TRF1 and TRF2 are related to the *Sacchromyces cerevisiae* Tbf1 protein, known as a yeast TTAGGG repeat-binding protein (52). Recently, it has been reported that Tbf1 is recruited to sites of HO-induced DSBs and promotes end resection at these sites (53). Our finding that Cdk activity is required for the recruitment of (pT371)TRF1 to sites of DSBs raises an interesting question as to whether the role of TRF2 and Tbf1 in DSB repair may be regulated by Cdk activity.

Although TRF1 is predominantly located at telomeres (10), we have previously reported that a fraction of endogenous TRF1 can also stably exist free of telomeres in the nucleus and that this pool of TRF1, but not telomere-bound TRF1, is phosphorylated at T371 by Cdk1 (16). Several lines of evidence suggest that it is this telomere-free pool of TRF1 that participates in DNA DSB repair. First, we did not detect any change in the level of telomere-bound TRF1 in response to IR through both analysis of differential salt extraction (Figure 1A) and chromatin immunoprecipitation (M. McKerlie and X.-D. Zhu, unpublished data), consistent with previous findings that DNA damage reagents do not release TRF1 from telomeres (54). Second, we have shown that overexpression of a mutant allele of TRF1 (TRF1-R425V) (16,32), which is defective in binding to telomeric DNA, promotes TRF1 recruitment to sites of DSBs, consistent with the notion that being telomere-free allows TRF1 to participate in DNA DSB repair. Third, we have shown that inhibition of the Cdk1 activity or the lack of TRF1 phosphorylation at T371 impairs TRF1 association with DSBs, whereas a phosphomimic mutation at T371 supports its recruitment to sites of DSBs. These results suggest that TRF1 needs to be not only free of telomeric DNA, but also to be phosphorylated at T371 by Cdk1 to facilitate HR-mediated DSBs repair.

We have shown that (pT371)TRF1 recruitment to sites of DNA damage is dependent on both the Mre11/Rad50/Nbs1 complex and BRCA1. TRF1 has been reported in a yeast two-hybrid assay to interact physically with Nbs1 (14); however, we have not been able to detect a physical interaction between TRF1 and Nbs1 when both of them were tagged and overexpressed in 293 T cells (T. R. H. Mitchell and X.-D. Zhu, unpublished data). It is possible that TRF1 interaction with Nbs1 may be transient in cells. It has been reported that TRF1 interacts with BRCA1 in a manner dependent on the Mre11/Rad50/Nbs1 complex (54). Whether coordination between Nbs1 and BRCA1 is needed to recruit (pT371)TRF1 to sites of DSBs requires further investigation.

BRCA1 and 53BP1 are known to compete with each other to direct the choice of DSB repair by either HR or NHEJ (5). Our finding that BRCA1 and 53BP1 play opposing roles in recruiting phosphorylated (pT371)TRF1 to sites of DSBs raises the question as to whether (pT371)TRF1 might be involved in regulating the choice of DSB repair, although TRF1 depletion or the lack of its phosphorylation at T371 only impairs HR- or SSA-mediated DSB repair and has little impact on NHEJ-mediated DSB repair. We have shown that the lack of TRF1 phosphorylation at T371 impairs the formation of IR-induced RPA foci, suggesting that (pT371)TRF1 is important for facilitating DNA end resection needed for HR. How (pT371)TRF1 may facilitate DNA end resection requires further investigation.

HR is regulated by Cdks (39,40), the activity of which promotes efficient DNA end resection to generate RPA-coated single-stranded DNA. Several DSB repair proteins, such as Nbs1 and CtIP, have been shown to be phosphorylated by Cdks and their phosphorylation either activates DNA end resection (39,40) or mediates the complex formation important for DNA end resection (55,56). Our work reveals TRF1 to be a new player in the regulation of DNA end resection by Cdk1, adding further complexity to this process essential for HR. Furthermore, we have shown that loss of phosphorylated (pT371)TRF1 sensitizes cells to Olaparib, a PARP inhibitor potent in killing HR-deficient cancer cells (17), raising the possibility that phosphorylated (pT371)TRF1 might be exploited for cancer therapeutics.

## SUPPLEMENTARY DATA

Supplementary Data are available at NAR Online.

## FUNDING

Ontario Early Researcher Award [ER07-04-157] and Canadian Institutes of Health Research [MOP-86620 to X.-D.Z.]. Ontario Graduate Scholarship (to M.M. and T.R.H.M.). Funding for open access charge: Canadian Institutes of Health Research.

*Conflict of interest statement*. None declared.

## Supplementary Material

Supplementary Data
