# Supplementary material for: Phosphorylated (pT371)TRF1 is recruited to sites of DNA damage to facilitate homologous recombination and checkpoint activation
Source: Nucleic Acids Res. 2013 Aug 30;41(22):10268–82. doi: 10.1093/nar/gkt775 (PMC3905873; doi:10.1093/nar/gkt775)
Supplement: Supplementary Data [file supp_41_22_10268__index.html]

Phosphorylated (pT371)TRF1 is recruited to sites of DNA damage to facilitate homologous recombination and checkpoint activation — Phosphorylated (pT371)TRF1 is recruited to sites of DNA damage to facilitate homologous recombination and checkpoint activation — Supplementary Data 

# Phosphorylated (pT371)TRF1 is recruited to sites of DNA damage to facilitate homologous recombination and checkpoint activation

## Supplementary Data

files

**Files in this Data Supplement:**

- Supplementary Data - pdf file
