# Supplementary material for: Phosphorylated (pT371)TRF1 is recruited to sites of DNA damage to facilitate homologous recombination and checkpoint activation
Source: Nucleic Acids Res. 2013 Aug 30;41(22):10268–82. doi: 10.1093/nar/gkt775 (PMC3905873; doi:10.1093/nar/gkt775)

SUPPLEMENTARY FIGURE LEGENDS

Figure 1. Phosphorylated (pT371)TRF1 forms damage-induced foci in cells treated with IR, camptothecin or etoposide. (**A**) Indirect immunofluorescence with anti-pT371 antibody. SV40-transformed GM637 skin fibroblasts were mock treated or treated with 12 Gy IR. Cell nuclei were stained with DAPI in blue. (**B**) Indirect immunufluorescence with anti-pT371 antibody. Telomerase-immortalized BJ (hTERT-BJ) cells were mock treated or treated with 12 Gy IR. Cell nuclei were stained with DAPI in blue. (**C**) Indirect immunofluorescence with anti-pT371 antibody on HeLaII cells treated with camptothecin or etoposide. Cell nuclei were stained with DAPI in blue. (**D**) Quantification of the percentage of HeLaII cells with five or more damage-induced (pT371)TRF1 foci. Standard deviation from three independent experiments are indicated. CPT: camptothecin; Etop: etoposide.

Figure 2. TRF1 carrying an amino acid substitution of R425V is defective in localizing to telomeres *in vivo*. IF-FISH with anti-Myc antibody (red) in conjunction with FITC-conjugated-$(C_3TA_2)_3$ PNA probe (green) was performed with HT1080 cells overexpressing either Myc-tagged wild type TRF1 or Myc-tagged TRF1-R425V. Cell nuclei were stained with DAPI in blue.

Figure 3. Overexpression of TRF1 carrying a nonphosphorylatable mutation of T371A impairs the activation of the G2/M checkpoint and fails to promote cell survival following the treatment with DSB-inducing agents. (**A**) Quantification of the percentage of cells stained positive for H3-pS10. HT1080 cells stably expressing the vector alone or various TRF1 alleles as indicated were mock treated or treated with 12 Gy IR. For each cell line, a total of 3000 cells were scored in blind. Standard deviations from three independent experiments are indicated. (**B & C**) Clonogenic survival assays following various doses of IR (B) or camptothecin (C). The colony forming assays were performed with HT1080 cells overexpressing the vector alone or various Myc-tagged TRF1 alleles as indicated. Standard deviations

from at least three independent experiments are indicated. (**D**) Western analysis of HT1080 cells overexpressing the vector alone or various Myc-tagged TRF1 alleles as indicated. Immunoblotting was performed with anti-Myc or anti-γ-tubulin antibody.

Figure 4. Depletion or loss of ATM impairs the formation of IR-induced (pT371)TRF1 foci. (**A**) Indirect immunofluorescence with anti-pT371 antibody on IR-treated HeLaII cells knocked down for ATM. Cell nuclei were stained with DAPI in blue. (**B**) Quantification of the percentage of cells with five or more IR-induced (pT371)TRF1 foci in HeLaII cells with or without depletion of ATM from (A). Standard deviation from three independent experiments are indicated. (**C**) Western analysis of HeLaII cells with or without knockdown of ATM. Immunoblotting was performed with anti-ATM, anti-pT371, anti-TRF1 or anti-γ-tubulin antibody. (**D**) Indirect immunofluorescence with anti-pT371 antibody on IR-treated ATM-deficient cells complemented with either the vector alone (GM16666) or ATM (GM16667). Cell nuclei were stained with DAPI in blue. (**E**) Quantification of the percentage of cells with five or more IR-induced (pT371)TRF1 foci from (D). Standard deviations from three independent experiments are indicated. (**F**) Western analysis of ATM-deficient cells complemented with the vector alone or ATM. Immunobloting was performed with anti-pT371, anti-TRF1 or anti-γ-tubulin.

Figure 5. The Mre11/Rad50/Nbs1 is required for the formation of IR-induced (pT371)TRF1 foci. (**A**) Indirect immunofluorescence with anti-ATM-pS1981 antibody. HeLaII cells pretreated with DMSO or Mirin were radiated with 12 Gy IR and then fixed 1 hr post IR. (**B**) Quantification of the percentage of cells with five or more IR-induced ATM-pS1981 foci (A). Standard deviations from three independent experiments are indicated. (**C**) Indirect immunofluorescence with anti-pT371 antibody. HeLaII cells pretreated with DMSO or Mirin were radiated with 12 Gy IR and then fixed 8 hr post IR. (**D**) Western analysis. Prior to 12 Gy IR, HeLaII cell extracts were treated with DMSO or Mirin. Immunoblotting was performed with anti-pT371, anti-TRF1 or anti-γ-tubulin antibody.

Figure 6. Indirect immunofluorescence with anti-pT371 antibody on IR-treated Nbs1-deficient cells (NBS-ILB1) complemented with the vector alone or various Myc-tagged Nbs1 alleles as indicated. Cell nuclei were stained with DAPI in blue.

Figure 7. The formation of IR-induced (pT371)TRF1 foci is impaired in the breast cancer cell line HCC1937. (**A**) Indirect immunofluorescence with anti-pT371 antibody on IR-treated MCF7 (expressing functional BRCA1) or HCC1937 (lacking functional BRCA1) cells. Cell nuclei were stained with DAPI in blue. (**B**) Quantification of the percentage of cells with five or more IR-induced (pT371)TRF1 foci from (A). Standard deviations from three independent experiments are indicated. (**C**) Western analysis of MCF7 and HCC1937 cell extracts with anti-pT371, anti-TRF1, or anti-γ-tubulin antibody.

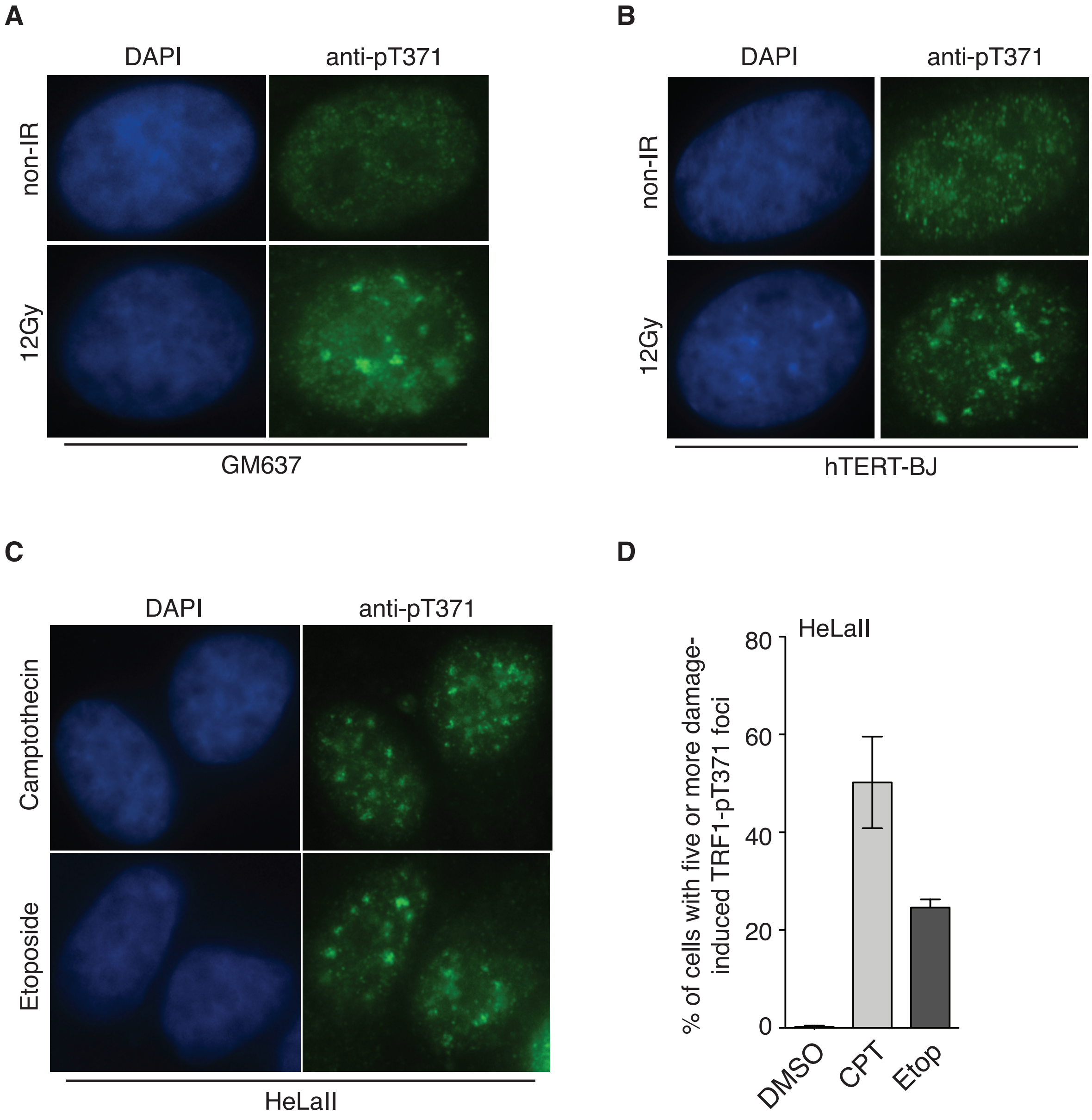
A
DAPI
anti-pT371
non-IR
12Gy
GM637
B
DAPI
anti-pT371
non-IR
12Gy
hTERT-BJ
C
DAPI
anti-pT371
Camptothecin
Etoposide
HeLaII
D
HeLaII
% of cells with five or more damage-induced TRF1-pT371 foci
0
20
40
60
80
DMSO
CPT
Etop

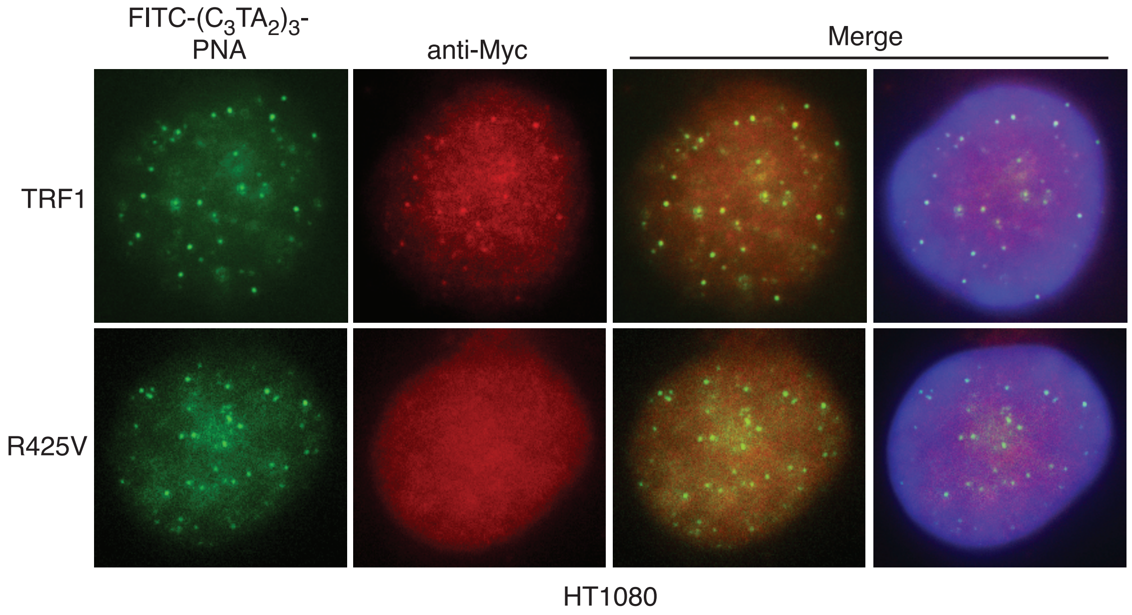
FITC-$(C_3TA_2)_3$-PNA
anti-Myc
Merge
TRF1
R425V
HT1080

Supplementary Figure 3 McKerlie et al.

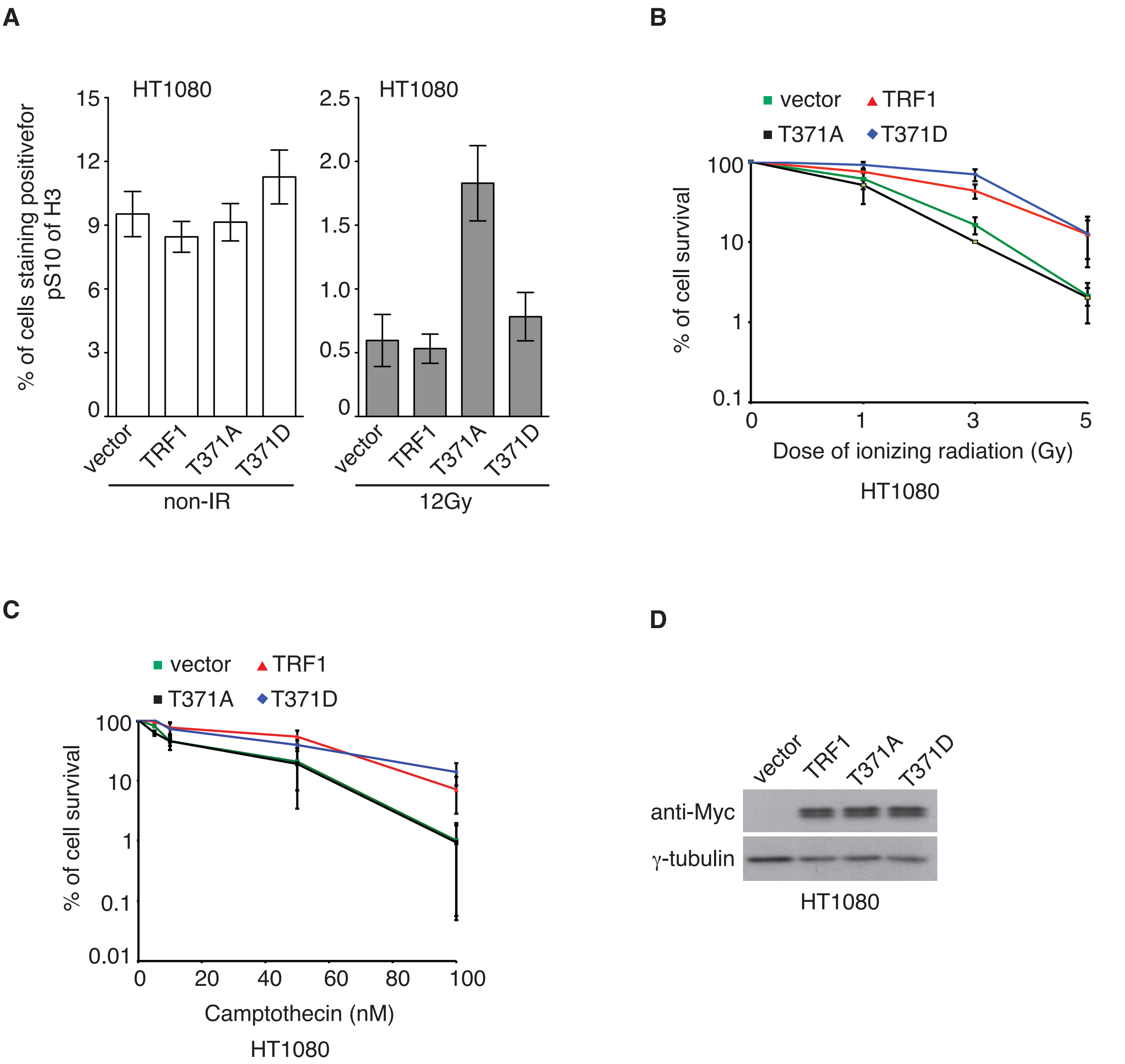

Supplementary Figure 4 McKerlie et al.

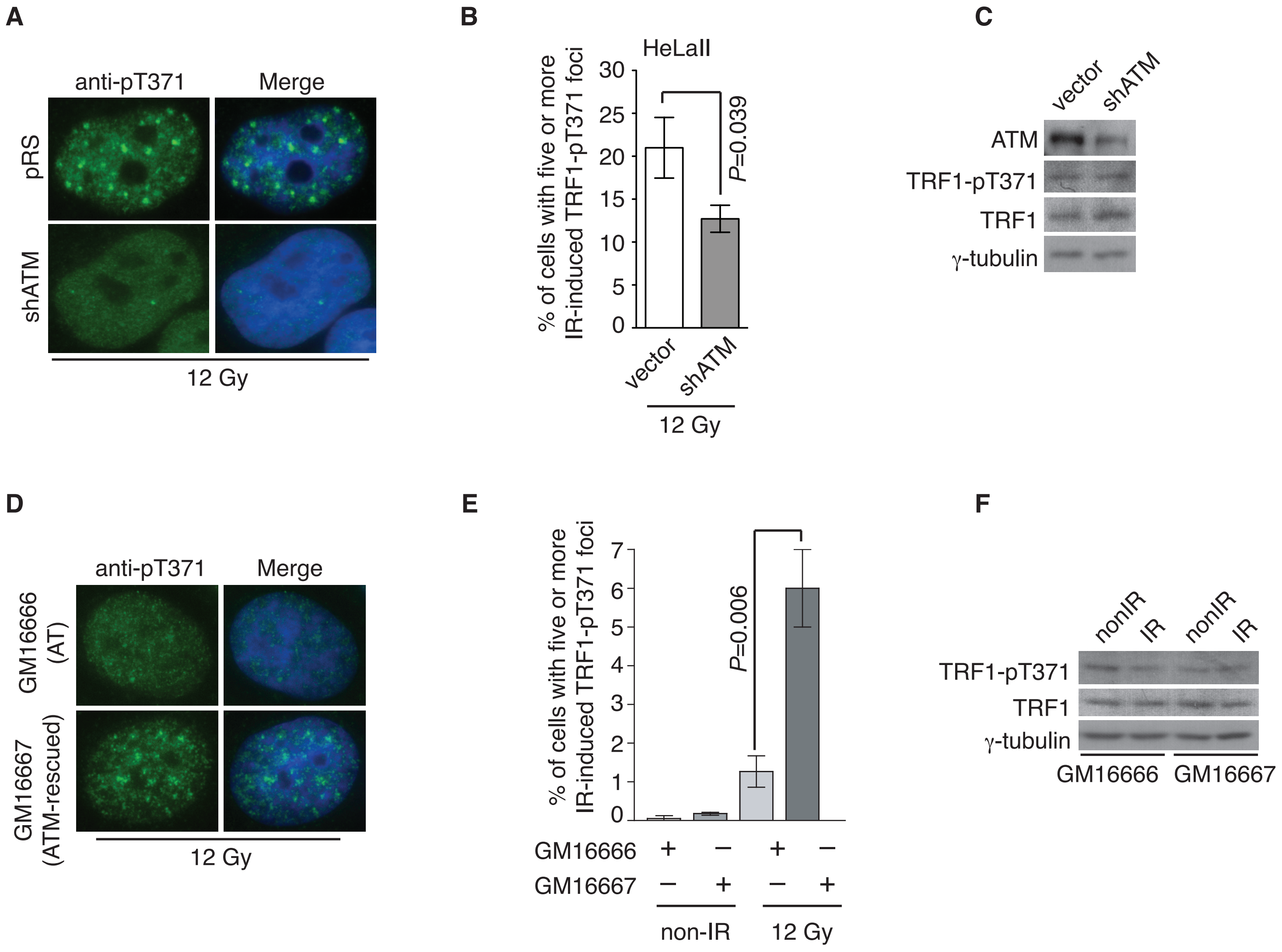

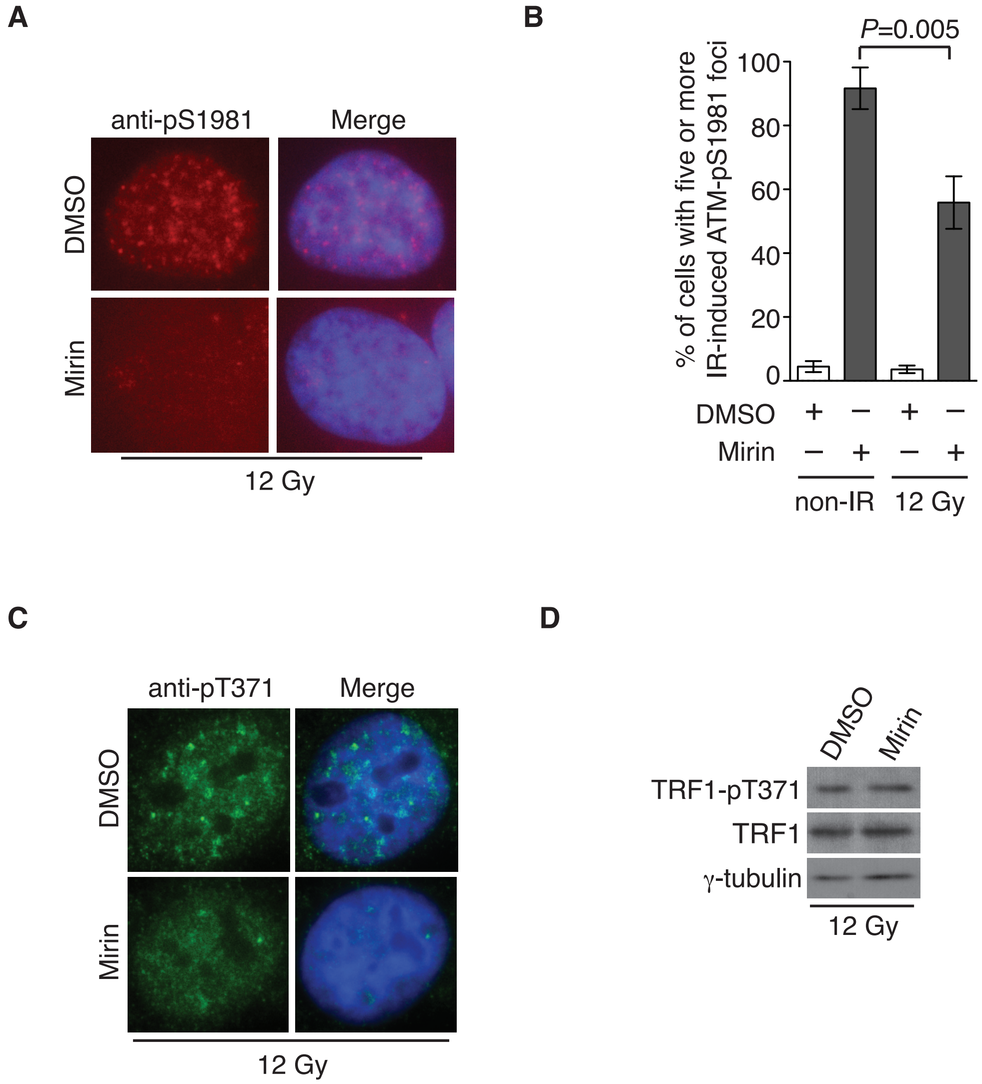
A
anti-pS1981
Merge
DMSO
Mirin
12 Gy
B
P=0.005
% of cells with five or more IR-induced ATM-pS1981 foci
100
80
60
40
20
0
DMSO + − + −
Mirin − + − +
non-IR
12 Gy
C
anti-pT371
Merge
DMSO
Mirin
12 Gy
D
DMSO
Mirin
TRF1-pT371
TRF1
γ-tubulin
12 Gy

Supplementary Figure 6 McKerlie et al.

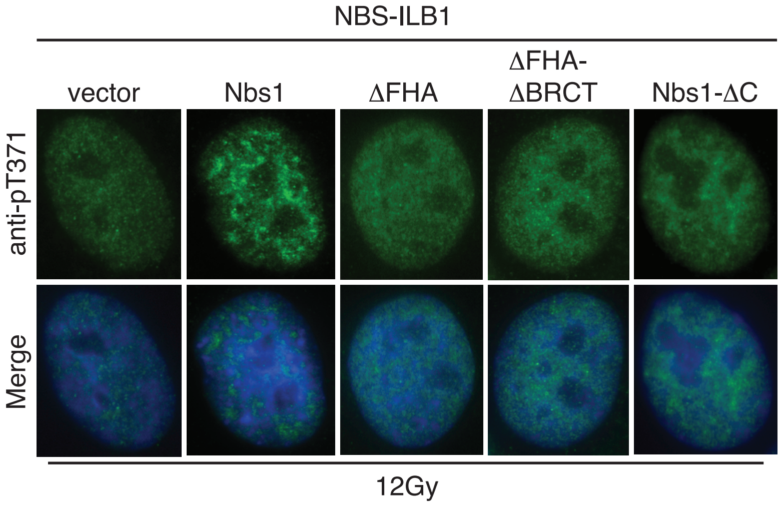

Figure 7 McKerlie et al.

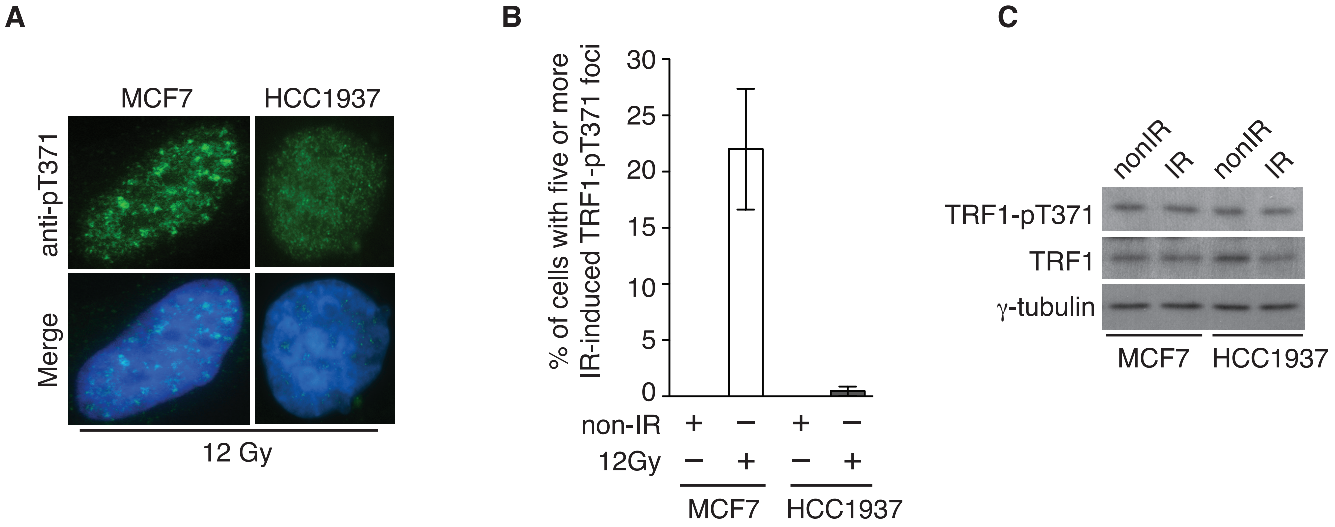

Supplement: Supplementary Data [file supp_gkt775_nar-01698-d-2013-File009.pdf]
